# Evolution and medicine: the long reach of "Dr. Darwin"

**DOI:** 10.1186/1747-5341-2-4

**Published:** 2007-04-03

**Authors:** Niall Shanks, Rebecca A Pyles

**Affiliations:** 1 Departments of History and of Philosophy, Wichita State University, Wichita, Kansas 67260, USA; 2 Honors College and Department of Biological Sciences, East Tennessee State University, Johnson City, Tennessee 37614, USA

## Abstract

In this review we consider the new science of Darwinian medicine. While it has often been said that evolutionary theory is the glue that holds the disparate branches of biological inquiry together and gives them direction and purpose, the links to biomedical inquiry have only recently been articulated in a coherent manner. Our aim in this review is to make clear first of all, how evolutionary theory is relevant to medicine; and secondly, how the biomedical sciences have enriched our understanding of evolutionary processes. We will conclude our review with some observations of the philosophical significance of this interplay between evolutionary theory and the biomedical sciences.

## Background: evolutionary medicine

In this review essay, we consider the interplay between evolutionary biology, on the one hand, and the biomedical sciences on the other. With certain exceptions (drug resistance in bacteria being an example) the ties between evolutionary theory and the biomedical sciences, though perhaps implicitly recognized, have until relatively recently, not been coherently articulated. In the last 15 years numerous books and articles have been written which have attempted to define "the new science of Darwinian medicine." Our aim in this review is thus to delineate the contours of this new branch of biomedical inquiry, and to draw out some of its philosophical significance.

The relationship between evolutionary biology and the biomedical sciences has not always been a comfortable one. Consider comparative physiology – a discipline with enormous implications for the biomedical sciences, given the importance of comparative animal studies. Over a decade ago, Burggrem and Bemis could lament:

Unfortunately comparative physiology traditionally has been, and continues to be, outside the framework of contemporary evolutionary biology, often embracing theories, positions or approaches that contemporary morphologists, evolutionary biologists, and geneticists have abandoned [[1]:193].

The relevance of evolutionary biology to medicine is also poorly understood by educated members of the public. Perhaps more disturbing are misunderstandings – and even a lack of understanding – of the relevance of evolutionary biology for medicine (in theory and in practice) by medical professionals. As Ewald has observed:

Evolutionary biology is so firmly integrated with the rest of biology that it is not possible to mark a boundary between them. But modern medicine has been a peninsula. It is broadly and firmly connected with most regions of biology. . . but has just a few thin bridges traversing the gulf to evolutionary biology. Knowledge about the evolution of antibiotic resistance is perhaps the best developed bridge between the disciplines. The discovery of the evolutionary basis for sickle cell anemia – protection against malaria – is another [[2]:7].

Ewald continues:

There are probably many reasons for the paucity of bridges. One stems from the inadequate appreciation of the pervasiveness of evolutionary principles. From secondary school through medical school, the fundamental relevance of evolution to all human life has often been ignored or even suppressed [[2]:7].

We believe it is important for the public, as consumers of medical services, and for medical practitioners themselves to have a greater appreciation of the medical implications of evolutionary biology. At its cutting edge, evolutionary biology has serious consequences for our understanding of human health and well-being – consequences that we ignore at our peril. The issues here will take us into the doctor's office and the hospital – places a long way away from the study of fossils of long-dead animals.

Evolutionary biology is a major research specialty in its own right, and a full review of the matters at hand would take many volumes. In this essay we focus on some key areas where evolution has had an impact on our understanding of medical phenomena. We begin with a discussion of some aspects of evolutionary biology.

## Evolution: Darwin and beyond

Much biology prior to Darwin was rooted in what may be termed *typological thinking*. Species represented fixed types, and though it was recognized that there could be variants on the theme of a given type (e.g., domestic dogs), it was the type itself, not the variants that were of crucial importance. Charles Darwin's great achievement [3] was to turn this idea on its head by emphasizing the importance of heritable variation in populations of interest, and to show how evolutionary mechanisms – natural selection in particular – could not only account for the exquisite adaptations seen in nature – adaptations hitherto attributed to providential intelligent design – but could also account for the origin of species – the very types that earlier generations had supposed were fixed and unchanging.

Part of Darwin's aim in his work on natural selection was to show how it was possible for populations of organisms, over successive generations, to *adapt *to problems confronting them in the environments with which they interacted. The classical Darwinian explanation of the evolution of organismal characteristics known adaptations rests on three basic ideas: (1) Populations of organisms show variation with respect to certain inherited characteristics of their members. (2) Individuals in such variable populations differ in rates of survival and reproduction by virtue of their characteristics, thereby manifesting differential reproductive success; and (3) The *heritable *characteristics that contribute to differential reproductive success will often be inherited by the progeny of successful individuals. In short, evolution occurs when different individuals leave behind different numbers of offspring. Over successive generations, other things being equal, those characteristics contributing to reproductive success will manifest themselves as adaptations. Following Sober [[4]:85], we may define adaptation as follows:

Characteristic *c *is an adaptation for doing task *t *in a population if and only if members of the population now have *c *because ancestrally there was selection for having *c *and *c *conferred a fitness advantage because it performed task *t*.

It should be noted that organisms have characteristics that are not, properly speaking, adaptations. Consider your blood. There was certainly selection for molecules capable of bearing oxygen. The redness of blood, however, is not an adaptation. It is simply a consequence of the chemistry of iron. Similarly, while there has been selection in the human lineage for large, problem-solving brains, the ability to do differential calculus is not an evolutionary adaptation – it is rather an accidental by-product of selection for other characteristics.

Darwin himself was aware of the distinction between the physical, abiotic environment and the biotic environment (predators, prey, pathogens and parasites, etc.). To this we may usefully add *culture *as a dimension of the biotic environment. Culture and a capacity for cultural evolution is not unique to the human species, yet humans have transformed the environment with which they (and other species) interact – and humans, along with other species of organisms, have in turn been transformed by the effects of cultural evolution. Cultural evolution is fast – just consider the changes that occurred in the course of the twentieth century – and it occurs within the lifetimes of long-lived organisms such as ourselves. As we will see below, cultural evolution has important medical implications. For organisms like us, with relatively long intervals between generations, rapid evolutionary responses to cultural changes are typically not possible, leading to the phenomenon of *environment discord*. For organisms with much shorter intervals between generations – every twenty minutes for bacteria such as *Staphylococcus aureus*, a major cause of wound infection – rapid, heritable, adaptive responses to such environmental/cultural products as antibiotics are not only possible, but have become a major medical problem. This is one of the major reasons why an understanding of the evolutionary phenomenon of host-parasite co-evolution is of vital importance.

Darwin knew virtually nothing about the mechanisms of inheritance and had precious little knowledge of organic chemistry (biochemistry was still a largely unformed intellectual fetus during his lifetime). After 1900, with the rediscovery of Mendel's insights about the particulate factors involved in the inheritance of characteristics, genetics emerged as a science in its own right. Of crucial importance here are events occurring from the 1920s through the 1950s – a period that gave rise to what historians of biology know as the *new evolutionary synthesis*. Here ideas about genetics were fused with ideas about evolution. The result was that population genetics – especially the study of the ways in which the relative frequencies of variant forms of genes (alleles) can change over successive generations – became the corner stone of modern evolutionary thought. In the course of this intellectual revolution, natural selection (resulting in adaptations) emerges as but one way in which allele frequencies can change. Other mechanisms that can shift allele frequencies include random genetic drift, gene flow (the effects of emigration and immigration), assortative mating, and a variety of linkage effects. As an understanding of bacterial evolution grew, it gradually became clear that, in addition to "vertical" gene transfers across the generations, there are occurrences of "horizontal" gene transfers (e.g., genes conferring resistance to various drugs can be exchanged between members of an extant population – there may even be cross-species horizontal transfers). Such transfers, when they occur, can permit extremely rapid evolution.

In the last quarter century enormous strides have been made as evolutionary biologists have learned the need to fuse their gene-based perspective on evolution with insights drawn from developmental biology. The resulting ideas – discussed under the rubric of evolutionary developmental biology – have come to constitute an intellectual revolution in their own right. The results of various genome projects have shown an enormous genetic similarity between humans, chimps, dogs and mice. At the level of the genes centrally involved in development (e.g., the so-called *Hox *genes), we are virtually identical. Notwithstanding this, humans and our evolutionary relatives are clearly very different types of organisms. It is now beginning to emerge that the key to understanding this diversity in the face of so much similarity is the study of gene regulation. For a crude analogy, two identical piano keyboards can play very different tunes – what matters is the order and timing with which the keys are played [see [5-10]].

As evolutionary biology has itself evolved, so too have its implications for the biomedical sciences and the practice of medicine. In the last twenty-five years, a growing number of evolutionary theorists have started to build bridges between evolutionary biology and the biomedical sciences [2,11]. This has culminated in the emergence of a new discipline called *Darwinian Medicine*. Darwinian Medicine is not offered as an alternative to existing branches of medical inquiry, but rather as a means of enriching our current understanding of biomedical phenomena [12]. It's a two-way street: as evolution enriches our understanding of medical phenomena, medicine enriches our understanding of evolutionary principles. For example, studies of the nature of humoral immunity [13,14] as well as cancer [15], have given evolutionary biologists valuable insights into the mechanisms of adaptive evolution as they shape the fates of populations of cells in multi-cellular organisms. Some of these examples are discussed later in this essay. We begin, however, with examples of the importance of evolutionary ideas to some of the biomedical sciences.

## The biomedical sciences: variation and species differences

Perhaps the most important consequence, historically, of a failure to appreciate the implications of evolutionary biology for the biomedical sciences, lies with the importance that evolutionary biologists place on variation, both within and between evolving populations of organisms. As noted by Burggrem and Bemis:

While comparative physiologists have made an art of avoiding the study of variation, such heritable variation nonetheless is the source of evolutionary changes in physiology as well as for all other types of characters [[1]:201].

Ignoring interspecific differences and intraspecific variation, there has been a historic trend for comparative physiologists to revert to pre-evolutionary typological thinking involving a focus on paradigm "model" species. Again, as observed by Burggren and Bemis:

Yet the use of "cockroach as insect," "frog as amphibian," or "the turtle as reptile" persists, in spite of clear evidence of the dangers of this approach. Not surprisingly, this type of comparative physiology has neither contributed much to evolutionary theories nor drawn upon them to formulate and test hypotheses in evolutionary physiology [[1]:206].

These problems can also be illustrated by a consideration of the importance of interspecific variation, intraspecific variation and gene regulation in the context of pharmacology and toxicology – where the focus is all too often on "mouse or rat as mammal" – and, in particular, as "human being."

In the United States, 14 to 16 million animals are used in biomedical research each year. The vast majority of mammals (85 to 90 %) employed in such research, aimed at benefiting humans, are rodents [16]. Primate species are not a significant part of the total partly because they are difficult and expensive to house, and partly because, in the case of such species as gorillas, chimpanzees, and orangutans, they are close to extinction.

How then, is evolutionary biology relevant to a discussion of the use of animals in biomedical research aimed at benefiting humans? In terms of the pattern of evolutionary relationships, the line leading to modern humans seems to have diverged from the lineage leading to modern rodents about 70 million years ago, thus representing an accrual of some140 million years of independent evolution. The lineage leading to modern mice seems to have diverged from that leading to modern rats some 17 million years ago. It is quite easy to conclude that rats and mice are more closely related to each other than either is to humans.

From a genetic point of view, the human genome project has revealed that the human genome consists of some 30,000 genes. The mouse genome is about the same size as the human genome [17]. Moreover, reflecting common ancestry, counterparts (or "orthologs") of many human genes have been identified in both mice and rats (notwithstanding differences in chromosomal arrangement). From the standpoint of genetic "base-pair similarity," humans, rats, and mice are remarkably similar. But the devil of genetic differences between individuals of a species, or the genetic differences between members of different species, lies in the details.

Mammals are diploid organisms, which means they have two sets of chromosomes, one from each parent. Such chromosomes in a diploid individual are said to be homologous because that they have the same pattern of genes along the chromosome. The location of a given gene on a chromosome is known as its *locus*. For a given locus, different versions of a gene – as the alleles – may exist in an individual (limited to two versions) and/or in a population (two or more versions). Such allelic variation generates variation with respect to the genotypes found in a population, and is thus a major source of genetic polymorphisms.

Though each individual has two alleles at a given locus (one from each parent), a large population of such individuals may exhibit several (more than two) alleles for a given gene. The various relative frequencies of alleles may be computer for any population. Different alleles typically have different biological properties. When these properties influence the reproductive success of the organisms bearing them, with the effect that different organisms in the population leave behind different numbers of offspring, then evolution occurs – over successive generations, the frequencies with which given alleles are found in the population changes. Certainly, allele frequencies can change for other reasons too, but this need not concern us here.

The main implication of evolutionary biology for our inquiries is the uncontroversial observation that in natural populations (whether of mice or humans), there is typically variation with respect to the alleles that are present. But typical laboratory populations of (say) mice are represented by highly inbred strains or varieties. The value of an inbred strain is supposed to lie in its relative genetic homogeneity. The hope is that individuals belonging to such strains should respond similarly when similarly stimulated (perhaps with drugs or toxins). The use of highly inbred individuals is a way to control for the real genetic variation in natural populations which can confound the results and conclusions of laboratory experiments. Thus, the problem of interspecies extrapolation from rodents to humans (where there are genetic similarities, but not genetic identities) is exacerbated by the fact that human populations will often not only contain alleles very different from those in rodent populations (where similar genes can be identified), but will also typically exhibit allelic variation that is absent in the (homogeneous) laboratory rodent populations used to model them. The "model" is confounded both by lack of identical (or even similar) biological properties of alleles and by lack of overall genetic variation.

Now apply the use of our "model" to a consideration of the biomedical study of drug and toxin metabolism. The enzyme system that plays an important role in xenobiotic (drug and toxin) metabolism is the cytochrome *P450 *system. Some 500 different *P450 *enzymes have been characterized by description of their DNA sequences, and members of a given species may carry 40–50 of these different enzymes [18]. For ease of reference, we will use "CYPs" as an abbreviation for the Cytochrome P450 enzyme group in the following discussion.

First, some terminology should be introduced. The CYPs represent a superfamily of genes and each gene (and its product enzyme) is named according to the similarity of its DNA sequence to other genes in the superfamily. The following example will help. Consider CYP 1A2. The first number designates the family the gene belongs to, which is determined on the basis of at least 40% sequence similarity. The letter following then designates a subfamily, determined on the basis of at least 59% sequence similarity. The last number identifies the specific gene (or enzyme). CYP 1A2 and CYP 3A4, for example, belong to different families within the CYP superfamily. By contrast, CYP 2C9 and CYP 2D6 belong to same family, but different subfamilies. We know also that each gene is composed of alleles, which may differ, so specific alleles are denoted by an asterisk and additional number. CYP 2D6*10 refers to a specific allelic variant (*10) of the CYP2D6 gene and so on.

### Human intraspecific variation

Within biomedical sciences, we all too readily speak of mice and humans as if all mice and all humans were the same. For many reasons, this is an error from an evolutionary perspective. In our current example, human CYP polymorphisms can manifest themselves in the form of intraspecific (i.e., individual) differences in drug metabolism. Two genes, CYP 2D6 and CYP 2C19 are particularly important since they affect how people metabolize approximately 25% of the drugs on the market [19].

Sipes and Gandolfi [20] observed that with respect to the antihypertensive agent debrisoquine, some 3 to 10 percent of Caucasians are poor metabolizers because they are homozygous for 2 nonfunctional alleles for CYP 2D6, the gene source of debrisoquine 4-hydroxylase enzyme. There appear to be more than 75 allelic variants of CYP 2D6 circulating in human populations [21].

Among these 75 variants, frequencies of the distribution of alleles vary among different ethnic populations: for example, individuals homozygous for the *10 allele have low CYP 2D6 gene activity and are found in nearly 20% of the Japanese population – a figure that differs from both Caucasian and Chinese populations [22]. Studies in molecular genetics indicate that actual cause of reduced activity of the CYP 2D6 gene is variable and complex. Causal factors range from single nucleotide polymorphisms in the coding sequences, to effective deletions of the gene itself, to polymorphisms that affect the splicing of CYP 2D6 [21]. But on the other side of the coin, there are rapid metabolizers with high CYP 2D6 gene activity, related to the fact they possess duplicates of the gene (some with as many as thirteen copies). High metabolizers with high CYP 2D6 gene activity require more than the standard doses of drugs to achieve therapeutic responses. It should be obvious that these important human differences could never have been revealed by nonhuman animal studies.

Consider the metabolism of a specific drug, such as the antiepileptic drug mephenytoin. More than 20% of the Japanese population are poor metabolizers (compared to about 3% of the Caucasian population [20]). Enzymes in the CYP 2C subfamily have been shown to be responsible for mephenytoin metabolism, with CYP 2C19 responsible for the main enzyme, (S)-mephenytoin 4'-hydroxlase [18]. Poor metabolizers appear to make a stable, but defective protein [20]. The presence of CYP 2C19*2 and *3 alleles account for 99% of poor metabolizers within oriental populations and 87% of Caucasian poor metabolizers.

These examples represent only a minute sample of what is known about polymorphisms with respect to the specific enzymes and substrates (drugs, in this case) mentioned. But they highlight the importance of paying attention to intraspecific variation when considering metabolic activity. Partly for these reasons, Collins has recently pointed out:

In the field of metabolism, as well as some segments of toxicity and efficacy, there has been a major shift from animal-derived data to human-based data. Except for comparative studies to assess interspecies differences, animal studies have declined in importance. Part of this shift is driven be an appreciation for the uncertainty in cross-species metabolic pathways. From the practical side, the well-organized, readily available supply of human tissue has fueled this shift [[23]:238].

The existence of intraspecific variation is but a foretaste of the biological problems confronting those who seek to use animals to model human biomedical phenomena. As Darwin observed in the *Origin of Species*:

As each species tends by its geometrical rate of reproduction to increase inordinately in number; and as the modified descendants of each species will be enabled to increase by as much as they become more diversified in habits and structure, so as to be able to seize on many and widely different places in the economy of nature, there will be a constant tendency of natural selection to preserve the most divergent offspring of any one species.

Hence, during a long continued course of modification, the slight differences characteristic of varieties of the same species, tend to be augmented into the greater differences characteristic of species of the same genus [[3]:108].

In other words, one effect of evolutionary processes in the formation of new species, is essentially to amplify the differences that existed in the varieties belonging to the common ancestor from which the new species descend in the course of evolutionary time. Thus, further bad news lies in the fact that interspecific variation is likely to be even more of a problem for the animal modeler than the already confounding intraspecific variation we have just discussed.

### Extrapolation between rodent species

As noted above, rats and mice are more closely related to each other than either is to humans. While intraspecific variation is important within rat and mouse (and human) populations – marked differences exist between different strains of mice and different strains of rats with respect to drug metabolism and susceptibility to diseases such as cancer. Interspecific extrapolation between rats and mice has proved to be no simple matter – rats are not particularly good models for mice! Thus as Hoffman has observed:

Correspondence between mouse and rat, the two most commonly used species in carcinogenicity tests, is not especially high. For 73 compounds evaluated by Tennant *et al*., the concordance between mouse and rat was 67%. Moreover, in an evaluative study by Griesemer and Cueto, only 44 of 98 agents that were carcinogenic in either rats or mice were carcinogenic in both species [[24]:216].

### Extrapolation from rodents to humans

There is an enormous literature on the problems associated with extrapolation from rodents to humans. We will briefly examine three examples to highlight the difficulties encountered in any such enterprise.

#### (a) Cancer

As aptly described in one of the leading textbooks in cell biology:

The mouse is the most widely used model organism for the study of cancer, yet the spectrum of cancers seen in mice differs dramatically from that seen in humans. The great majority of mouse cancers are sarcomas and leukemias, whereas more than 80% of human cancers are carcinomas – cancers of epithelia where rapid cell turnover occurs. Many therapies have been found to cure cancers in mice; but when the same treatments are tried in humans they usually fail [[25]:1347].

There are 26 *known *human carcinogens (the list of probable carcinogens is somewhat longer). Of these 26 carcinogens, humans are exposed to seven by inhalation. Do carcinogenicity assays involving rodents convey information about human risk? Two decades ago, Salsburg observed of the 26 *known *human carcinogens:

Most of these compounds have been shown to cause cancer in some animal model. However, many of the successful animal models involve the production of injection site sarcomas or the use of species other than mice or rats. If we restrict attention to long-term feeding studies with mice or rats, only 7 of the 19 human non-inhalation carcinogens (36.8%) have been shown to cause cancer. If we consider long-term feeding or inhalation studies and examine all 26, only 12 (46.2%) have been shown to cause cancer in rats or mice after chronic exposure by feeding or inhalation. ... On the basis of probability theory we would have been better off to toss a coin [[26]:64].

Should we be alarmed if a substance induces cancer in rats or mice? Probably not, especially in view of the fact that rodents have exhibited carcinogenic responses to 19 out of 20 substances suspected of being non-carcinogenic in humans [27]. Thus, the data available today do not support the assumption that these particular animal "models" actually are models for human carcinogenicity studies.

#### (b) Diabetes

Differences with respect to gene regulation may be illustrated by the following example. It has been shown that xenobiotics induce transcription of certain families of CYPs by activating nuclear receptors. CYP 3As, for example, are regulated by the pregnane X receptor (PXR). Studies have been performed on human and mouse orthologs of PXR. Moore *et al*. commented upon the results of these studies as follows:

However, comparison of PXR from four different species shows that this receptor has diverged considerably in the course of evolution. The human, rabbit and rodent PXR are all roughly equally divergent and share only ~70% amino acid identity. This divergence in PXR is an important component of cross-species differences in the regulation of CYP3A expression by xenobiotics [[28]:15126].

Species differences are not just associated with evolution of the structures of CYP enzymes, they are also associated with evolution in the molecules that regulate the expressions (on, off, or actual amount) of the genes coding for those enzymes as well. The regulatory role of PXR is indeed medically significant. The following provides an excellent example of this significance.

The CYP 3A family is particularly important in the context of xenobiotic metabolism because, as Jones *et al*., have noted:

The CYP 3A gene products are among the most abundant of the monooxygenases in mammalian liver and intestine. In humans, CYP 3A4 is involved in the metabolism of more than 50% of all drugs as well as a variety of other xenobiotics and endogenous substances, including steroids [[29]:27].

One drug that is of interest in this regard is troglitazone (marketed as Rezulin^®^) and used in the treatment of type-II diabetes. Troglitazone was removed from the market in the U.S. in March 2000. Despite the fact that it had been shown to be safe and effective in rodent studies [29], more than 65 people died (two-thirds were women), and many other required liver transplants as a result of Rezulin^® ^toxicity. In clinical trials involving a total of 2500 human subjects, about 2% showed alanine aminotransferase (ALT) levels more than 3 times the upper limit of normal. ALT levels this high are an indicator of active liver disease (see [[30]:114–119] for details of how Rezulin^® ^came to market on the FDA "fast track").

The class of drugs to which troglitazone belongs was developed using rodent models of insulin resistance, but without prior knowledge of the cellular target [29]. It is now known that troglitazone achieves its therapeutic effects by binding to the PPARγ nuclear receptor. But the concentrations required to activate PPARγ also activate the PXR nuclear receptor in humans – something it did *not *do in rats and mice [29]. Thus, one immediate consequence of this interspecific difference is that human patients taking troglitazone experienced increased CYP 3A4 activity. Jones *et al*., comment:

Our data showing that troglitazone activates human PXR at concentrations similar to those required to activate PPARγ provide an explanation for its interactions with other drugs, including oral contraceptives. Interestingly, the relative lack of activity of troglitazone on the mouse or rat PXR may explain why these effects were not reported in animal toxicology studies. Additional studies will be required to determine whether PXR also plays a role in the hepatotoxicity observed with troglitazone. In this regard it is interesting that that the PXR ligand rifampicin has also been associated with hepatotoxicity in humans [[29]:36].

Recently, it has been argued that the increased CYP 3A4 activity associated with troglitazone activation of human PXR results in the metabolism of troglitazone to a reactive quinone which has been hypothesized as the cause of hepatotoxicity [31]. Examples like this could be multiplied for our discussion here. However, the point is made that at the molecular level of life there are medically significant differences between species. These differences may arise from evolved differences in catalytic activity of enzymes, from evolved differences in the regulation of gene expression, or even as by-products of interactions created by the introduction of xenobiotics, never "seen" by nature or evolution.

The consequences of the belief that humans and rodents are the same molecular animal dressed up differently can be (and have been) catastrophic. As Goldstein recently put it in an editorial in the *New England Journal of Medicine*:

One of the most striking features of modern medicines is how often they fail to work. Even when they do work, they are often associated with serious adverse reactions. Indeed adverse reactions to drugs rank as one of the leading causes of death and illness in the developed world [[32]:553].

### Endocrinology and Public Policy

Observations of species differences in the context of comparative endocrinology have led at least some observers to give serious consideration to evolution's consequences. Thus Hart commented:

It has proved heuristically useful in studies on estrogens. . . to adopt the unifying concept that species differences in estrogen toxicity mirror species differences. . . in estrogen endocrinology. The poor predictiveness of animal studies for humans thus becomes comprehensible in terms of interspecies variations in endocrinology [[33]:213].

This matter is very urgent because it has become clear that a large number of substances in the environment have impact on estrogenic, androgenic and thyroid hormone activity. The US Environmental Protection Agency's (EPA) *endocrine disruptor study program *will be employed to examine these issues with a view to human safety and well-being.

But problems have been uncovered concerning the rodent strains selected to evaluate the human risk. As Spearow and Barkley have commented on the results of recent research:

. . . studies have revealed a tremendous amount of genetic variation in susceptibility to endocrine disruption by oestrogenic agents between strains of rats and mice. These studies have shown that the highly prolific, large litter size selected CD-1 mice and Sprague-Dawley rats most commonly used for product-safety testing are much more resistant to oestrogenic agents than other strains examined [[34]:1027].

CD-1 mice are at least 16-fold more resistant than other strains of mice (including B6) to compounds that cause inhibition of testis weight, a measurement used as an indicator of androgen activity. CD-1 mice are 126-fold more resistant than B6 mice to inhibition of sperm maturation by estradiol [34]; the authors add:

The most favored EPA rodent model for endocrine disruptor testing, the Sprague-Dawley rat, is also more resistant than other strains to inhibition of testis weight by (DES) diesthylstiboestrol. Furthermore Sprague-Dawley rats are highly resistant while Fisher-344 rats are highly sensitive to oestradiol, DES or BisPhenol A induced hyperprolactinaemia, uterine and vaginal hypertrophy, hyperplasia, mucous secretion and c-Fos induction [[34]:1027].

Since evolution tends to amplify differences between populations after the cessation of gene flow, short of highly fortuitous convergent evolution (nowhere demonstrated), it is unlikely that interspecific differences will be less than those observed among different strains of the same species of rodent. If not highly unlikely, it would, at the very least, be imprudent to assume in such important studies.

A good illustration of the problem here lies in the fact that the rodents selected for the study of endocrine disruption in humans have been selected for the pragmatic virtue of large litter sizes. But, as researchers have noted:

We should realize that an animal that has been selected for high fecundity regulates reproduction quite differently than unselected individuals. Furthermore, these highly prolific strains tend to be quite precocious, with many "immature" CD-1 females showing vaginal opening and elevated uterine weights even in response to the 0 dose control treatment. Such precocious sexual development and the resulting elevation of ovarian oestrogen production complicates, if not limits, the use of strains previously selected for high prolificacy for detecting osestrogenic activities in intact uterotropic assays [[34]:1028].

Lying at the heart of an evolutionary view of animal populations is variation. Variation exists both within and between populations. With the cessation of gene flow between populations, initial variation between ancestral populations of a given species can be expected to be amplified in successive generations. Alleles rare in one such population may become common in another, and so on.

What then is to be done in the light of observations such as these? Constructive suggestions for the future course of medicine already exist that are harmonious with evolutionary theory, and emanate from such branches of biomedical science as pharmacogenetics and pharmacogenomics (a branch of pharmacology using genome-wide techniques to study inherited differences with respect to drug response). The long-term goal of pharmacogenomics is that of therapy tailored to an individual patient – therapy that reflects the uniqueness of the individual as a member of an evolving population. As has been observed by Evans, et al.,

The potential is enormous for pharmacogenomics to yield a powerful set of molecular diagnostic methods that will become routine tools with which clinicians will select medications and drug doses for individual patients. . . Genotyping methods are improving so rapidly that it will soon be simple to test for thousands of single nucleotide polymorphisms in one assay [[35]:546–547].

What are we to do in the meantime? Population studies with respect to drug metabolism are already providing clinically relevant insights. Again, an observation by Evans, et al.,

. . . a specific genotype may be important in determining the effects of a medication for one population. . . but not for another; therefore, pharmacogenomic relations must be validated for each therapeutic indication and in different racial and ethnic groups. Remaining cognizant of these caveats will help ensure accurate elucidation of genetic determinants of drug response and facilitate the translation of pharmacogenomics into widespread clinical practice [[35]:547].

While the specter of "*race-based" *medicine is sure to raise hackles (see relevant discussions [32,36-38]), we already know of many *statistical *associations of certain, metabolically significant, allelic variants with certain racial and ethnic groups. That is to say, two populations may differ with respect to the relative statistical frequencies of certain allelic variants. Many of these associations are simply results of long past events, such as natural or human-created barriers that separated populations. Until such a time as individualized therapy is possible, matters of ethnicity ought to be one of the factors taken into account in a rational discussion of the course of drug therapy, as these matters currently are recognized and used as a factor in genetic counseling. The pre-evolutionary, typological "one therapy fits all," possibly rooted in "Caucasian (male) as human" model, requires serious critical scrutiny.

## Host-parasite co-evolution

Lying at the heart of modern evolutionary theory, as it impinges directly on medicine, is the concept of host-parasite co-evolution. Indeed, the study of human responses to infectious, parasitic agents such as bacteria and viruses is one of the few places where evolutionary theory has had a major impact on medical theory and practice. As we will see, however, it has not been "plain sailing" and basic misunderstandings of evolution's implications for these matters are still prevalent.

Paul Ewald has done much to clarify matters in this regard by critically analyzing the views common, albeit erroneous, in the medical community, including (a) that evolution works for the benefit of the species; and (b) that parasitism and the resulting diseases are steps on the road to a state of happy co-existence [2]. According to this author [2], Rene Dubos claimed in 1965, "Given enough time a state of peaceful coexistence eventually becomes established between any host and parasite." In 1972 Lewis Thomas observed that, "Disease usually represents the inconclusive negotiations for symbiosis. . . a biological misrepresentation of borders." [2]. And as late as 1989 Paul Hoeprich could claim, "The ideal of parasitism is actually commensualism" [2].

The claim that evolution works for the *benefit of the species*, though still common outside of evolutionary circles, has been substantially abandoned by professionals in the field of evolutionary biology in favor of a thoroughly genocentric view of evolution. As we saw earlier, evolution occurs because different individuals leave behind different numbers of offspring – offspring carrying a proportion of alleles identical by descent to those found in the parents. In the case of diploid organisms, the offspring receive (on average) 50% of their alleles from each parent. In asexual, clonal species (the proportion will be 100%, barring horizontal genetic transfers (a non-trivial assumption for bacterial species). One way or another, it is alleles that travel down the generations.

Evolution has no eye for the future – it does not operate with a view to the attainment of teleological ends or typological goals. In particular, neither evolution nor the presence of particular characteristics can properly be characterized as a steady march of progress toward traits beneficial (in our minds) to the species as a whole. As Ewald has observed:

Scientist's errors can often be traced to the belief that natural selection will favor what is best for the long-term stability and survival of the species. In fact natural selection is powerless to favor such long-term survival when it runs counter to short-term competitive gains. By the time the long-term benefits would be accrued, the individuals that could provide them would have vanished from the species by competition. This misunderstanding owes much to the catchiness of the phrase "survival of the species," which emphasizes the species rather than the competitors within the species" [[11]:xiv].

Such misconceptions are intimately linked to the mistaken view that evolution in the context of host-parasite relationships is a steady march to a state of "benign coexistence," and hence to mistaken expectations about the evolution of virulent pathogens and parasites. Again, as Ewald pointed out:

Natural selection favors characteristics that increase the passing on of the genes that code for the characteristics. If more rapid replication of a virus inside of a person leads to a greater passing on of the genes that code for that rapid replication, then replication rate will increase even if the more rapid growth of the virus population within a person causes the person to be severely ill, or leads to an overall decrease in the numbers of the virus among people, or hastens the eventual extinction of the virus [[2]:4].

This phenomenon can be explored in the context of *within-host selection*. Diseases differ with respect to virulence. For most of us the common cold is a nuisance. The rhinovirus works its evolutionary mischief by keeping its host mobile – and hence typically in contact with other susceptible persons who in turn help with the reproduction and dispersal of the virus. By contrast, highly virulent strains of malaria (e.g., that caused by *Plasmodium falciparum*), rapidly immobilize the host and kill millions of human each year.

Since malaria is propagated by biting mosquitoes, the parasite pays no penalty for an immobilized host – especially one too weak to swat the insect vector. Moreover, simultaneous infection with different strains of *P. falciparum *with varying degrees of virulence creates a competitive environment. In such a situation, those strains that attain highest concentrations in the host's blood in the least amount of time (thereby wreaking havoc on the host) are those most likely to be sucked up by biting mosquitoes, who then spread the progeny of these virulent strains to other susceptible hosts [2]. Another example concerning the illogic of obligate evolution to a state of benign co-existence, is provided by Nesse and Williams:

What good would it do a liver fluke to restrain itself so as not to harm the host if that host is about to die of shigellosis? The fluke and the *shigella *are competing for the same pool of resources within the host, and the one that most ruthlessly exploits that pool will be the winner. Likewise, if there is more than one *shigella *strain, the one that most effectively converts the host's resources to its own use will disperse the most progeny before the host dies [[12]:57].

That millions of parasitic progeny die with the host does not matter. What matters is differential reproductive success on the part of individual parasites. These are population level phenomena that occur within a single host, but still result in the passing on of characteristics that aid effective dispersal of offspring into fresh hosts. A typological goal of benign coexistence simply does not exist in these instances.

No discussion of host-parasite co-evolution would be complete without at least a nod in the direction of the evolution of drug resistance by bacteria and viruses. Antibiotics are *differential poisons *– in this case they are more toxic to bacteria than they are to us. But bacterial populations show variation with respect to susceptibility to a given antibiotic. A given clinical dose of antibiotics should so damage the bacterial population that the few survivors should be dealt with by the host's own immune system. But if the full course of treatment is not followed (or the patient resorts to unsupervised self-treatment), the antibiotic becomes an agent of selection favoring bacteria whose genetic constitution can tolerate the antibiotic in question. The result? Offspring of resistant bacteria will inherit alleles coding for these characteristics and these populations will increase. Continued treatment with such an antibiotic will require higher doses – a process that cannot continue indefinitely, since patient toxicity will eventually become an issue. The remedy is to move on to a new antibiotic, and the whole process may repeat itself, sometimes with a similar outcome. Unfortunately, the situation with resistant bacteria is even worse, since individual bacteria also can transfer alleles conferring drug resistance horizontally to other members of their own population, as well as to members of other bacterial species that may be present in the host.

The situation described here has been observed in viral populations, with Human Immunodeficiency Virus (HIV) being a case in point. HIV is an RNA retrovirus that exhibits poor replicative fidelity. In effect, the virus replicates itself with the aid of the host's own cells, but does so with such lack of precision that a viral particle may produce many variants. These variant "offspring" may differ with respect to susceptibility to the hosts' immune surveillance or to anti-viral drugs, creating populations of "new" viral particles with different properties. Nesse and Williams observe:

A single infection, after years of replication, mutation and selection, can result in a diverse mixture of competing strains of the virus within a single host. The predominant strains will be those best able to compete with whatever difficulties must be overcome (e.g., AZT or other drugs). They will be the ones that most rapidly divert host resources to their own use – in other words, the most virulent [[12]:57].

HIV is not the only panic-generating virus in the news. Much worry is being devoted to avian flu and the possibility of another flu pandemic on the scale of the Spanish flu of 1918. Some words of evolutionary caution are called for even here.

The influenza virus particle displays molecules on its surface that can be recognized by the immune system. Different strains of influenza can be identified by their possession of variants of these molecules. Of particular interest are H-type molecules (versions of hemagglutinin) and N-type molecules (versions of neuraminidase). The Spanish flu of 1918 was caused by an H1N1 virus, in contrast to the avian flu currently in the news, which is an H5N1 virus. In 1976, a strain of flu with the H1N1 marker reappeared – causing much panic among flu experts. Was the panic justified? Arguably not, for as Ewald has observed:

The H1N1 marker had been present on dangerous viruses, but there was no reason to think that it *made *the viruses dangerous – with its high mutation rate, the influenza virus can generate tremendous variation within a matter of weeks while still retaining the H1N1 marker [[11]:23].

While an obsession with such "marker" molecules can be highly misleading, the evolutionary questions run to a deeper level of analysis. We have just seen that parasites and pathogens differ with respect to strategies for reproduction and dispersal – some keep their hosts mobile, some succeed by immobilizing their hosts. An evolutionary analysis considers the virus in ecological context. The conditions that led to the differential reproductive success of the highly virulent Spanish flu of 1918 were somewhat unique – in particular, consideration has to be given to the crowded, unsanitary conditions that existed in the trenches at the Western front during WW1, along with the confinement of flu victims to crowded barracks, and subsequently to over-crowded hospitals. The mere existence of a dangerous virus does not amount to much – unless conditions exist that favor its differential replicative success and subsequent dispersal. Commenting critically of approaches adopted by influenza experts, Ewald points out:

. . . they still confuse the sources of variation – the mutation and recombination of genes – with the process of evolution by natural selection. And they still confuse similarity of hemagglutinin and neuraminidase molecules among different virus strains with similarities in the virulence of these strains. . . By failing to investigate the selective processes that favor increased or decreased virulence of virus strains, experts still run the risk of spending too much time and too many resources in attempts to block a 1918-type pandemic, and too little time on how to deal with the more immediate threats [[11]:25].

The long reach of evolutionary biology into the field of medicine does not stop here, for evolutionary principles can be observed with respect to populations of specialized cells that are found normally in multi-cellular organisms such as ourselves.

## The immune system and cancer

Multi-cellular organisms are composed of cells belonging to a wide variety of types. Careful studies of the behavior and dynamics of some of these cellular populations have revealed that we ourselves are being shaped and influenced by adaptive evolutionary principles during the course of our individual lives. Here we discuss two examples to illustrate the application of basic evolutionary principles to these medically significant phenomena.

### (a) Humoral Immunity

The immune response concerns the reaction of the body (*self*) to invasion by foreign substances (non-*self*). In the context of humoral immunity, foreign substances (perhaps viruses or bacteria or parts thereof) known as *antigens *stimulate B-lymphocyte cells to produce molecules known as *antibodies*. Antibodies react with antigens to tag them for further immunological responses. The details of the antibody-antigen reaction are instructive for present purposes.

A specific antibody, carried by a B-lymphocyte, is capable of "recognizing" (by chemical binding) a limited range of antigenic molecular shapes. For a given antigen, some antibodies never bind, some do rarely, and some will bind to the antigen virtually every time they encounter it. There is enormous variation and diversity in the antibody population – the system is capable of recognizing more than 10^8 ^antigen shapes. Once an antibody binds to an antigen, the B-cell can receive a second signal from a T cell. This combination of signals stimulates the particular B-lymphocyte to divide (mitosis) and make daughters of itself. The proportion of these particular B-cells thus increase in the lymphocyte population, which then, in turn, create more of the appropriate antibody. The B-lymphocyte population displays variation and, depending on selective antigenic binding and signals from other lymphocytes, differential reproductive success relative to those B-lymphocytes that failed to bind to the current invader.

Some B-lymphocytes become factories for the production of large numbers of antibodies to fight the current infection. But other successful B-lymphocytes remain in circulation in the body, providing the immune system with a memory of that particular antigen shape. This phenomenon explains why the immune response on subsequent re-infection is faster than the initial response. These binding characteristics of the descendents of the original successful B-lymphocytes are thus genuine Darwinian adaptations, in this case for immunological function. Indeed, Parham has observed:

At some point this century the experimental biologists, in an echo of Henry Ford, divorced themselves intellectually from the evolutionary biologists. This artificial and regrettable separation remains with us today. For the immunologists it was always a sham, for the very foundations of their subject are built upon stimulation, selection and adaptive change. Now we see clearly the immune system for what it is, a vast laboratory of high speed evolution. By recombination, mutation, insertion and deletion, gene fragments are packaged by lymphocytes, forming populations of receptors that compete to grab hold of antigen. Those that succeed get to reproduce their progeny, if antibodies, submit to further rounds of mutation and selection. There is no going back and the destiny of each and every immune system is to become unique, the product of its encounters with antigen and the order in which they happen. This all happens in somatic tissues, in a time frame of weeks and is perhaps too vulgar, too fast, for traditional tastes to be even called evolution [[13]:373].

Having a fast, adaptive immune system is clearly advantageous in a world where we are confronted with rapidly evolving pathogens and parasites. But, as we observed earlier, evolution occurs with no eye to the future. This can be illustrated through a consideration of the pathological phenomenon of cancer.

### (b) Cancer

In multi-cellular organisms, such as ourselves, there is a sort of "social contract" between cells of various specialized types (liver cells, kidney cells, etc). These cells are, for the most part, genetically identical. Kidney cells differ from liver cells primarily with respect to differential patterns of gene activation, not the genes themselves. Somatic cells of various specialized types cooperate (and ultimately perish with the death of the organism) so that the specialized reproductive cells (gametes) can get genes identical by descent into the next generation. Cancer cells can be thought of as outlaws that violate the multi-cellular social contract. They replicate at the expense of their neighbors and ultimately at the expense of the organism bearing them.

The formation of a cancer cell is typically a multi-step process in which several mutant alleles must be acquired. The probability of a given cell becoming a cancer cell is small, but there are billions of cells – by analogy, the probability that you win the State lottery is small, but when millions of tickets have been sold, it is likely that somebody will win. Cancer cells begin as mutant versions of healthy cells, and they are cells that have acquired the ability to activate their own reproduction, producing almost identical clones. The reproductive process is not perfect, and the progeny of the initial cancer cell typically constitute a population of cells displaying variation with respect to heritable characteristics. The descendents of these cells themselves acquire mutations and eventually some may acquire the ability to migrate to new locations, thereby departing from the confines of their cellular origins. The end result is metastatic cancer. Untreated, and barring spontaneous remission, unrestrained cellular proliferation, with or without metastasis, typically brings about the failure of critical organ systems and death.

In the treatment of cancer using chemotherapy, an all too familiar evolutionary saga plays itself out. Chemotherapeutic agents are differential poisons that target speedily replicating cancer cells. Unfortunately, they also can affect other speedily replicating healthy cells (such as epithelial cells), which is why chemotherapy can have such awful side-effects. If you are lucky, the chemical agent eliminates all the cancer cells. Alas, quite often treatment reduces the cancer cell population to a few hardy survivors while giving the appearance of remission. But this small, now selectively hardy population may gradually repopulate the patient. The resultant growing population bears the genetic inheritance that enabled the cancer cells to survive the initial therapeutic assault – a genetic inheritance resulting in the evolution of drug resistance. Now the oncologist is required to try new agents, until they, too, are rendered ineffective through the adaptive evolution of populations of the cancer cells in question. As Greaves has observed:

. . . cancer. . . is a form of evolution played by the same Darwinian ground rules as apply to evolution in general and particularly for asexually propagating species. The essential game plan is progressive diversification by mutation within a clone, coupled with selection of individual cells on the basis of reproductive and survival fitness, endorsed by their particular gene set. Its evolution on the fast track [[15]:39].

## Conclusion

It is clear that evolutionary biology has an enormous potential to enrich our understanding of biomedical phenomena. It is also clear that the study of biomedical phenomena can greatly enrich our understanding of evolutionary processes. These observations should be of relevance to biological and biomedical investigators and educators. Moreover, the examples drawn from immunology and oncology show that the human body itself is a laboratory for fast evolution. This fact has significant philosophical implications for the philosophy of science, especially as it relates to the nature of explanations in the biological sciences.

Much of this review has been devoted to ways in which evolutionary biology can enrich our understanding of biomedical phenomena. However, the study of biomedical phenomena shows the need to rethink some aspects of evolutionary biology. Traditional Darwinists draw a sharp distinction between mechanistic explanations on the one hand, and evolutionary explanations on the other. Thus, in dealing with the question, "What is biology?" the great evolutionary biologist Ernst Mayr observed:

When we try to answer this question, we find that biology actually consists of two rather different fields, mechanistic (functional) biology and historical biology. Functional biology deals with the physiology of all activities of living organisms, particularly with all cellular processes, including those of the genome. These functional processes ultimately can be explained purely mechanistically by chemistry and physics [[39]:24].

But the story does not end so simply; Mayr continues:

The other branch of biology is *historical biology*. A knowledge of history is not needed for the explanation of a purely functional process. However, it is indispensable for the explanation of all aspects of the living world that involve the dimension of historical time – in other words, as we now know, all aspects dealing with evolution. This field is evolutionary biology [[39]:24].

Mayr observes that the most frequently asked question is mechanistic (functional) biology is "how?" whereas the most frequently asked question in evolutionary biology is "why?" He adds, "To truly appreciate the nature of biology one must know the remarkable difference between these two branches of biology" [39].

Focusing their attention on the contours of the new science of Darwinian medicine, traditional Darwinists Nesse and Williams distinguish between two types of causes that are medically relevant (and thus require two different types of causal explanation):

Consider heart attacks. Eating fatty foods and having genes that predispose to atherosclerosis are major causes of heart attacks. These are what biologists call proximate ("near") causes. We are more interested here in the evolutionary causes that reach further back to why we are designed the way we are. In studying heart attacks, the evolutionist wants to know why natural selection hasn't eliminated the genes that promote fat craving and cholesterol deposition. Proximate explanations address how the body works and why some people get a disease and others don't. Evolutionary explanations show why humans, in general, are susceptible to some diseases and not to others [[12]:6].

The distinction is between mechanistic explanations that answer "how" questions, and evolutionary explanations that answer "why" questions. Evolutionary explanations are typically viewed as long-term, historical explanations (one might have to consider the entire course of human evolution, for example), whereas mechanistic explanations are immediate – and for many purposes, essentially ahistorical.

It is true that looking at medical phenomena from the standpoint of traditional Darwinism typically means taking a historical perspective – and as we have seen above, it certainly has a legitimate role to enrich our understanding of biomedical phenomena. It is also true that traditional Darwinists recognize that rapid evolution is possible for organisms with short generation times, such as viruses and bacteria – organisms where the relevant history may concern events occurring over the course of a few months. But we now see that traditional Darwinism is only a part of the Darwinian medical story. Consideration also needs to be given to the role of Darwinian explanations of biomedical phenomena occurring in the life-cycles of animals – including humans.

The examples we have presented concerning the role of Darwinian explanations in the realms of immunology and oncology show that it is not easy to draw a sharp distinction between mechanistic explanations and evolutionary explanations. For phenomena in the domain of humoral immunity and oncology, important aspects of the mechanistic explanation involve a rapid evolutionary explanation. In this way the study of biomedical phenomena shows the need for a critical reassessment of generalizations about the nature of biological explanation that have been forthcoming from traditional Darwinists.

## Competing interests

The author(s) declare that they have no competing interests.

## Authors' contributions

NS and RAP participated equally in the research, writing and revisions for this manuscript. NS and RAP both read and approved the final manuscript.
